# Evaluation of room temperature (30°C to 35°C) lyophilized vaccine with radio inactivated *Mannheimia haemolytica* whole cells isolated from infected sheep

**DOI:** 10.14202/vetworld.2022.1261-1268

**Published:** 2022-05-23

**Authors:** Sahar Ahmed, Waleed Abdelgaber Nemr, Walaa Awad Ahmed Mohamed, Amany Mohamed Mohamed, Mohamed Abd El-Fatah Mahmoud

**Affiliations:** 1 Department of Cell Biology, Biotechnology Research Institute, National Research Centre, 12622 Dokki, Giza, Egypt; 2 Department of Radiation Microbiology, National Centre for Radiation Research and Technology, Egyptian Atomic Energy Authority, Egypt; 3 Central Laboratory for Evaluation of Veterinary Biologics, Abbassia, Cairo, Egypt; 4 Department of Parasitology and Animal Diseases, Veterinary Research Institute, National Research Centre. Dokki Giza, Egypt

**Keywords:** enzyme-linked immunosorbent assay, gamma radiation, interferon-gamma, interleukin 4, *Mannheimia haemolytica*, vaccine

## Abstract

**Background and Aim:**

Vaccines are one of the important tools for fighting diseases and limiting their spread. The development of vaccines with high efficacy against diseases is essential. Ionizing radiation is the method used for the preparation of the irradiated gamma *Mannheimia haemolytica* vaccine. The study aimed to measure the metabolic activity and electron microscopic examination of the irradiated bacterial cells and immunological efficiency of different preparations of the irradiated *M. haemolytica* vaccine.

**Materials and Methods:**

The irradiated vaccines were prepared in three forms at a dose of 2×10^9^ colony-forming unit (CFU) (irradiated *M. haemolytica*, trehalose irradiated *M. haemolytica*, and trehalose lyophilized irradiated *M. haemolytica*). The formalin-killed vaccine was prepared at a dose of 2×10^9^ CFU. Scanning electron microscopy was used to determine the difference between the non-irradiated bacterial cells and the bacterial cells exposed to gamma radiation. The metabolic activity of the irradiated bacterial cells was measured using the Alamar blue technique. Rabbits were divided into five groups (control, vaccinated groups with the formalin-killed vaccine, irradiated bacterial cells without trehalose, trehalose irradiated bacteria, and trehalose lyophilized irradiated bacterial cells). The rabbits were subcutaneously inoculated twice in 2-week intervals. Enzyme-linked immunosorbent assay, interferon-gamma (IFNγ), and interleukin 4 (IL4) assays were used to evaluate the vaccines’ immunological efficiency in rabbits.

**Results:**

The metabolic activity tests showed that the bacterial cells exposed to gamma radiation at the lowest lethal dose have metabolic activity. The difference in the metabolic activity between preparations of the irradiated bacterial cells varied according to the cell concentration and incubation time. The highest level of metabolic activity was 8 h after incubation in the nutrient broth medium compared with 4 and 18 h. The scanning electron microscopy of irradiated bacterial cells showed a cavity at the bacterial cell center without rupture of the surrounding cell membrane compared to the non-irradiated bacterial cells. The antibody level in the groups vaccinated with the different preparations of the irradiated bacterial cells was high compared with the control and formalin-killed vaccine groups. The level of the IFNγ showed an increase after the second dose in the group vaccinated with irradiated bacterial cells without trehalose compared with the other groups. The IL4 level in the vaccinated groups with the irradiated bacterial cells without trehalose, irradiated bacterial cells with trehalose, and trehalose lyophilized irradiated bacterial cells were at a high level when compared with the formalin-killed vaccinated group and control group after the second inoculation.

**Conclusion:**

The irradiated *M. haemolytica* vaccine provides a wide range of humoral and cellular immunity. This study showed high immunological efficiency in rabbits inoculated with the irradiated *M. haemolytica* vaccine that was shown in the high levels of antibodies (IFNγ and IL4) compared with the group treated with the formalin-killed vaccine. The second dose of irradiated *M. haemolytica* vaccine is an immune booster that gives the irradiated vaccine a long-acting immunological efficiency.

## Introduction

The preservation of livestock and their sustained development requires many important factors. The most important of these are controlling the diseases and limiting their spread to preserve livestock with high productivity. This contributes to preserving food security and eliminating the food gap and poverty resulting from the lack of animal production. Vaccines are one of the important tools in fighting diseases and limiting their spread. The inactivated pathogen vaccine (IPV) is a non-viable pathogen that preserves its immunogenic structure and presents a non-hazardous form of the pathogen in the immune system. It simulates an infection event and then triggers an adaptive immune response against this pathogen as a protective action in the body. Moreover, this type of vaccine is comparably stable and safer than a live-attenuated vaccine, because IPV has a lower risk, especially for pregnant women, elderly, vulnerable, or immunodeficient individuals [1-3].

There are many methodologies for inactivating pathogen infectivity, primarily based on heat, chemical, or radiation treatment [2]. The chemical-treated IPV with formalin or the heated preparation has insufficient immunological efficiency [3] compared with the inactivation of IPV by ionizing radiation in vaccinated animals [2-12]. The immunological advantage of the ionizing radiation vaccine is due to the ionizing radiation destroying cellular nucleic acids at a non-effective dose on the protein structure. This preserves the metabolic activity and the antigenicity of the pathogen cell as an effective immunogen without the ability for replication. However, the common challenge is how to reduce the indirect damaging effect of ionizing radiation on the cellular proteins, which is caused by generating destructive free radicals from water molecules during irradiation [13].

The study aimed to use the ionizing radiation method to prepare an irradiated gamma *Mannheimia haemolytica* vaccine to study the metabolic activity and electron microscopic examination of the irradiated bacterial cells and immunological efficiency of different preparations of the irradiated *M. haemolytica* vaccine.

## Materials and Methods

### Ethical approval

The study was approved by Animal Ethics Committee of National Research Centre, Egypt.

### Sample collection and bacterial isolation

The criteria for the selected samples was the initial diagnosis of respiratory infection. The samples were collected from the pneumonic lung tissue of the slaughtered sheep. Swabs of the lung tissue specimens were inoculated onto MacConkey’s agar, blood agar containing 5% blood sheep, and nutrient broth plates. The colonies were assessed morphologically and tested biochemically by inoculation of the peptone water grown culture of each isolate in 1% glucose, sucrose, sorbitol, mannitol, fructose, dulcitol, lactose, salicin, arabinose, and maltose and were then incubated aerobically at 37°C for 72 h. Indole, oxidase, catalase, and nitrate reduction tests were performed according to Songer and Post [14].

### Bacterial cells irradiation protocol

The identified *M. haemolytica* cells were inoculated into the nutrient broth for 18-24 h at 37°C in a shaking incubator. Bacterial cells were collected by centrifugation at 1467 xg for 15 min. Bacterial pellet was re-suspended in 1 ml fresh broth medium before irradiation carried out in the National Center for Research and Radiation Technology, Cairo, Egypt. Bacterial cells were irradiated undercooling (the irradiator chamber was filled with ice packs during the irradiation process) at a dose equal to 20 KGy using Gammacell 220 Cobalt-60 Irradiator Facility; the dose rate at the time of this experiment equals 0.97 KGy/h [2]. In order to prepare the lyophilized irradiated vaccine, *M. haemolytica* bacterial cell suspension was mixed with trehalose (final concentration=10% W/V) before lyophilization and then irradiated at a dose of 20 kGy.

### Detection of metabolic activity of the irradiated bacterial cells

The irradiated bacterial cell viability was tested using the Alamar blue kit (Bio-Rad, USA), following the manufacturer’s instructions. The non-radiated bacterial cells and irradiated bacterial cells without trehalose, trehalose irradiated bacteria cells, and trehalose lyophilized irradiated bacterial cells were inoculated in nutrient broth medium at different cell concentrations (1×10^8^, 1×10^9^, and 1×10^10^ CFU). The Alamar blue assay was measured at a wavelength of 550 and 630 nm as a reference after 4, 8, and 18 h.

### Electron microscopic examination

A scanning electron microscope was used to determine the differentiation between the non-irradiated bacterial cells and the bacterial cells exposed to gamma radiation at the National Research Center. The collected isolates were fixed in glutaraldehyde before being negatively stained with 2% phosphotungstic acid and then examined on a 400 mesh grid using a scanning electron microscope (Joel EM 100 CX II at 30 Kv., Japan) [15].

### Vaccine preparation

The irradiated vaccine dose was evaluated as per Ahmed *et al*. [2]. The 2×10^9^ CFU/mL cell concentration has a high immune response compared with lower responses in 4×10^9^ CFUmL as well as other doses (1×10^5^, 1×10^6^, 1×10^8^, and 1×10^9^ CFU unpublished data). The irradiated vaccines were prepared in three forms at a dose of 2×10^9^ CFU mL (irradiated bacterial cells vaccine, irradiated bacterial cells 10% trehalose vaccine, and lyophilized irradiated bacterial cells 10% trehalose vaccine). The lyophilized irradiated vaccine was stored at room temperature (30°C to 35°C) The formalin-killed vaccine was prepared at a dose of 2×10^9^ CFU mL according to Selim *et al*. [16].

### Evaluation of the immunological efficiency of the different irradiated vaccines in rabbits

#### Animals

White New Zealand rabbits, 7-8 weeks old, were barrier-bred, unvaccinated, and free of a variety of pathogens. Rabbits were allowed for 1 week period of acclimatization following their arrival at the vivarium. The rabbits were individually housed in stainless steel cages and slatted bottoms that did not contain bedding. The rabbits were allowed *ad libitum* access to fresh tap water by water bottles and were fed a balanced commercial feed. The total number of rabbits used in the study was 30, and their weight ranged from 1.250 to 1.550 Kg.

#### Experimental design

There were five groups with six rabbits per group. The rabbits were inoculated 2 times in a 2-week interval at 2×10^9^ CFU dose of prepared vaccine. The control group was injected with phosphate buffer saline (PBS) (pH=7.4), Group 1 (G1) was inoculated with the formalin-killed vaccine, Group 2 (G2) was inoculated with an irradiated bacterial cell vaccine without trehalose, Group 3 (G3) was inoculated with an irradiated bacterial cell vaccine with 10% trehalose, and Group 4 (G4) was inoculated with lyophilized irradiated bacterial cells 10% trehalose after resuspension in PBS. The rabbits were subcutaneously vaccinated twice with 2-week intervals.

#### Blood samples collection

Two milliliters of blood were collected from each rabbit on days 0, 7, and 14 after the first vaccine inoculation and on days 7 and 14 after second vaccine inoculation.

#### Enzyme-linked immunosorbent assay (ELISA)

An ELISA assay was used after the first and second inoculations. The ELISA plates coated with the sonicated antigen 100 μL of 1:2 diluted antigen (IgG, Promega, France) in carbonate-bicarbonate buffer were incubated at 4°C for overnight. The coated ELISA plates were decanted and washed 3 times with the washing buffer and dried with a clean towel. The non-specific antibody binding sites were blocked using 1% bovine serum albumin in PBS (pH 7.4) by adding 100 μL/well. The plates were reincubated at 37°C for 30 min then decanted, washed, and dried. One hundred microliters of diluted tested serum were added to the coated well. Each serum sample was duplicated, including the control positive and negative sera and the blank control. The control positive was supplied from the ELISA unit at the Veterinary Research Institute, Egypt. The plates were covered and incubated at 37°C for 1 h. The plates were decanted and washed 3 times using the washing buffers. Then, 100 μL of the diluted conjugate 1:2 was added to all wells. The plates were covered and incubated at 37°C for 1 h. The plates were decanted and washed 3 times using the washing buffer solution. Then, 100 μL of the substrate o-phenylenediamine (OPD) was added for 15 min at 37°C. The reaction was stopped (by adding 25 μL/well of 1.25 mol sulfuric acid) and the plate optical density (OD_450_) was read [17].

#### Interferon gamma (IFNγ) and interleukin 4 (IL4) assays

IFNγ and IL4 were measured twice 1 week after the first and second inoculations for all groups, according to the manufacturer’s instructions of each kit (Kingfisher-Biotch. Inc, USA) at a wavelength of 450 nm.

### Statistical analysis

Calculation of metabolic activity of the bacterial cells was followed by the guideline provided by Bio-Rad. The metabolic activity was measured as a percentage difference between treated and control cells at a wavelength of 550 and 630 nm.

Metabolic activity data were statistically analyzed using the Statistical Package for the Social Sciences software program version 26 (IBM Corp., NY, USA). The data for the effect of different treatments and concentrations were analyzed using a two-way analysis of variance. Data for IL4, IFNγ, and antibody levels were analyzed using a one-way analysis of variance and an independent sample t-test. Significance between means was measured using the Duncan’s *post hoc* test at p≤0.05.

## Results

### Biochemical identification

The bacterial colonies identified as *M. haemolytica* were beta-hemolytic on blood agar. They were Gram-negative, short rods by microscopic morphology. In addition, they were indole negative, catalase, oxidase, and nitrate reduction positive and fermented sugars like lactose.

### Metabolic activity of the irradiated bacterial cells

**Figure-1:**
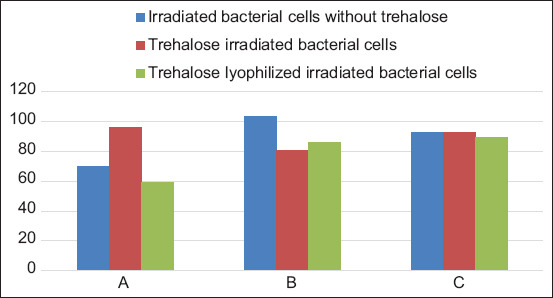
Metabolic activity of the irradiated bacterial cells after 8 h from culture inoculation. A=Bacterial cell concentration 10^10^, B=Bacterial cell concentration 10^9^, and C=Bacterial cell concentration 10^8^.

The results of the metabolic activity test showed that the bacterial cells exposed to gamma radiation at the lowest lethal dose have metabolic activity. The difference in the metabolic activity between preparations of the irradiated bacterial cells varied according to the cell concentration and incubation time. The highest level of metabolic activity was 8 h after incubation in the nutrient broth medium compared with 4 and 18 h. The metabolic activity was calculated as the percentage difference between the treated and control cells. Table-1 and Figure-1 depict the level of metabolic activity in the prepared irradiated bacterial cells 8 h after incubation at different bacterial cell concentrations. The metabolic activity of irradiated bacterial cells was not significant at cell concentrations 10^8^ and 10^9^ for irradiated bacterial cells without trehalose, 10^8^ and 10^10^ for trehalose irradiated bacterial cells, and 10^8^ and 10^9^ for trehalose lyophilized irradiated bacterial cells. The irradiated bacterial cells without trehalose showed a high metabolic activity of 103.33±1.42 compared with other irradiated bacterial cells at different concentrations and preparations. The lowest metabolic activity was observed in trehalose lyophilized irradiated cells of 59.45±0.32 at a cell concentration 10^10^

**Table 1:** Metabolic activity of the irradiated bacterial cells after 8 h from culture inoculation.

Group	Concentration	Metabolic activity
Irradiated bacterial cells without trehalose	10^8^	92.70±9.19^a,b^
	10^9^	103.33±1.42^a^
	10^10^	70.01±4.44^c,d^
Trehalose irradiated bacterial cells	10^8^	93.02±7.20^a,b^
	10^9^	80.64±1.33^b,c^
	10^10^	96.10±5.14^a,b^
Trehalose lyophilized irradiated bacterial cells	10^8^	89.58±2.15^a,b^
	10^9^	85.75±2.95^a,b,c^
	10^10^	59.45±0.32^d^

Mean values with small letters (a,b,c, and d) in the same column significantly at p≤0.05

### Electron microscopic examination

**Figure-2:**
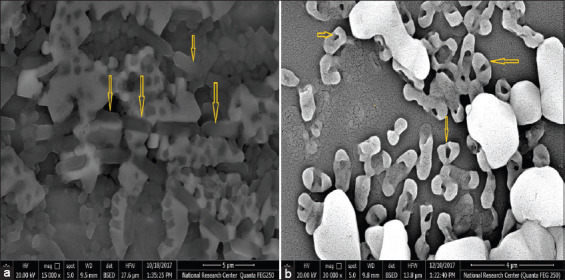
Scanning electron microscope photograph of the irradiated cells (a) and gamma irradiated cells of *Mannheimia* *haemolytica* (b), arrows indicate the difference of rod-shaped bacterial cells with the native structure (in the viable case) versus the opened structure after the exposure to the lowest lethal dose of gamma radiation.

Figure-2a and b show the difference between the non-irradiated bacterial cells and the bacterial cells exposed to the lowest lethal dose of gamma radiation. The irradiated bacterial cells showed a cavity at the bacterial cell center without rupturing the surrounding cell membrane (Figure-2b) compared with the non-irradiated bacterial cells (Figure-2a). The image represents the effect of gamma radiation on the deoxyribonucleic acid (DNA) molecule that destroyed the genetic material in the bacterial cells.

### Evaluation of the immunological effect of different forms of the irradiated vaccine in rabbits

#### ELISA antibodies assay

**Figure-3:**
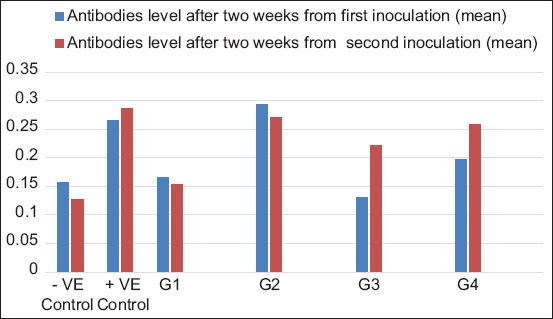
ELISA antibody level in rabbit groups after the first and second inoculations. ELISA=Enzyme-linked immunosorbent assay, G1=Formalin-killed vaccine, G2=Irradiated vaccine without trehalose, G3=Trehalose irradiated vaccine, and G4=Trehalose lyophilized irradiated vaccine.

The ELISA antibody levels in the different groups after the first inoculation dose showed a significant increase in the antibody level for G2 compared with the control group (Table-2 and Figure-3). After the second inoculation dose, the antibodies of groups G2, G3, and G4 showed significant differences (0.271±0.02, 0.221±0.01, and 0.258±0.02, respectively) compared with the control group (0.127±0.08) (Figure-3). The formalin-killed vaccine group had no significant difference compared with the control group after both inoculation doses (0.166±0.03 and 0.154±0.01 and 0.156±0.04 and 0.127±0.08, respectively).

**Table 2:** ELISA antibody level in rabbit groups after the first and second inoculations.

Group	After 2 weeks from the first inoculation (Mean±SD)	After 2 weeks from the second inoculation (Mean±SD)
−ve Control	0.156±0.04	0.127±0.08
+ve control	0.265±0.00	0.286±0.0
G1	0.166±0.03	0.154±0.01
G2	0.293±0.01*	0.271±0.02*
G3	0.171±0.10	0.221±0.01*
G4	0.198±0.02	0.258±0.02*

ELISA=Enzyme-linked immunosorbent assay, G1=Formalin-killed vaccine, G2=Irradiated vaccine without trehalose, G3=Trehalose irradiated vaccine, and G4=Trehalose lyophilized irradiated vaccine,

* the significant increase in the antibody level compared to the control at p*≤*0.01.

#### IFNγ assay

**Figure-4:**
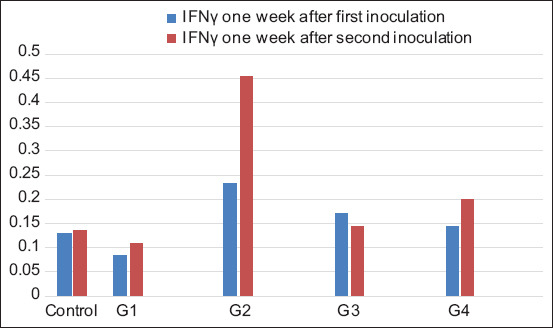
IFN level in rabbit groups after the first and second inoculations. G1=Formalin-killed vaccine, G2=Irradiated vaccine without trehalose, G3=Trehalose irradiated vaccine, and G4=Trehalose lyophilized irradiated vaccine.

The results of the IFNγ level between the first and second inoculations showed a significant difference in the groups vaccinated with formalin-killed vaccine (0.084±0.00 and 0.11±0.00, respectively) and irradiated vaccine without trehalose (0.232±0.09 and 0.454±0.08, respectively). In contrast, the control and other vaccinated groups (G3 and G4) had no significant differences between the two inoculations (Figure-4 and Table-3).

**Table 3:** Mean values of IFNγ level in rabbit groups after the first and second inoculations.

Group	IFNγ 1 week after the first inoculation (Mean±SEM)	IFNγ 1 week after the second inoculation (Mean±SEM)
Control	0.129±0.02	0.136±0.01^b^
G1	0.084±0.00^B^	0.11±0.00^A.b^
G2	0.232±0.09^B^	0.454±0.08^A.a^
G3	0.170±0.04	0.145±0.02^b^
G4	0.144±0.39	0.20±0.03^b^

IFNγ=Interferon gamma, G1=Formalin-killed vaccine, G2=Irradiated vaccine without trehalose, G3=Trehalose irradiated vaccine, and G4=Trehalose lyophilized irradiated vaccine. The small letters (a, b, and c) in the same column significantly at p*≤*0.05. The capital letters (A and B) in the same row significantly at p*≤*0.05

The results of the IFNγ level in the different groups after the first inoculation dose showed no significant differences between groups. However, after the second inoculation, the G2 group had a significant difference (0.454±0.08) compared with the groups inoculated with the formalin-killed vaccine, trehalose irradiated, trehalose lyophilized irradiated vaccine, and the control group (0.11±0.00, 0.145±0.02, 0.20±0.03, and 0.136±0.01, respectively).

#### IL4 assay

**Figure-5:**
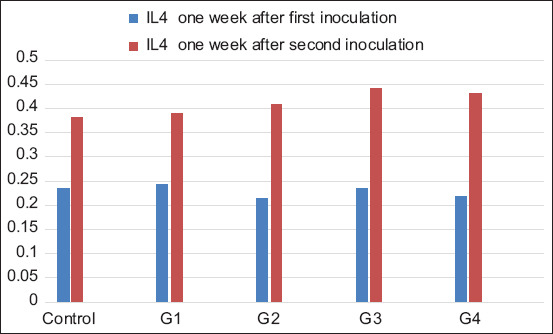
IL4 level in rabbit groups after the first and second inoculations. G1=Formalin-killed vaccine, G2=Irradiated vaccine without trehalose, G3=Trehalose irradiated vaccine, and G4=Trehalose lyophilized irradiated vaccine.

IL4 levels between the first and second inoculations showed significant differences in vaccinated and control groups (Figure-5 and Table-4). After the first inoculation, the results of the IL4 level between groups showed no significant differences. However, after the second inoculation, the groups inoculated with the irradiated vaccine without trehalose, trehalose irradiated, and trehalose lyophilized irradiated vaccines were significant (0.408±0.01, 0.440±0.02, and 0.431±0.02, respectively) when compared with the formalin-killed vaccinated and control groups (0.388±0.01 and 0.381±0.01, respectively). Moreover, the trehalose irradiated vaccine group had a high level of IL4 secretion compared with other irradiated vaccine groups.

**Table 4:** Mean values of IL4 level in rabbit groups after the first and second inoculations.

Group	IL4 1 week after the first inoculation (Mean±SEM)	IL4 1 week after the second inoculation (Mean±SEM)
Control	0.235±0.01^B^	0.381±0.01^A,c^
G1	0.242±0.02^B^	0.388±0.01^A,b,c^
G2	0.212±0.01^B^	0.408±0.01^A,a,b,c^
G3	0.236±0.02^B^	0.440±0.02^A,a^
G4	0.219±0.02^B^	0.431±0.02^A,a,b^

IL4=Interleukin 4, G1=Formalin-killed vaccine, G2=Irradiated vaccine without trehalose, G3=Trehalose irradiated vaccine, and G4=Trehalose lyophilized irradiated vaccine. The small letters (a, b, and c) in the same column significantly at p*≤*0.05. The capital letters (A and B) in the same row significantly at p*≤*0.05

## Discussion

Gamma irradiation is a highly effective technique applied to ensure the biological control of pharmaceutics and food supplies, along with the decontamination of dangerous organisms to enable their safe handling. Using this method, the development of vaccines based on radiation technology provides safe and highly effective immunity, removes chemical contaminants, and penetrates pathogens to damage the DNA or RNA [3,4].

Evaluation of the humoral immune effect in the vaccinated rabbits by ELISAs showed that the G1 group inoculated with the formalin-killed vaccine had the lowest level of antibody production compared with the other vaccinated groups, whereas G2 inoculated with the irradiated vaccine without trehalose had a high significant level of antibodies when compared with the control group after the first and second inoculations. The antibody level in G3 and G4 groups showed increases after the second inoculation dose with a significant difference compared with the control group (Figure-3 and Table-2). The results suggested that the irradiated vaccines increased the antibody levels, and the second dose is an immune booster. The increases in the level of antibodies in rabbit groups inoculated with different preparations of irradiated vaccines could be related to the metabolic activity recorded for the irradiated bacterial cells. The bacterial metabolic activity is an essential component of the disease process that shapes the systematic infection of the pathogen in the host [18,19]. *M. haemolytica* outer membrane has important immunogens such as *serotype 1-specific antigen*, *OmpA*, *OmpP2*, and *OmpD15*; these genes act to enhance immune protection in the host and stimulate the immune receptors to produce an acquired immunity that increases the level of antibodies and gives a wide range of immune protection [20,21]. The antibody results from the irradiated vaccine confirmed the results from Ahmed *et al*. [2,4].

The cellular immunity results suggested that the irradiated vaccine has a broad immune response where IFNγ, or type II interferon, is a cytokine that is critical for innate and adaptive immunity against viral, some bacterial, and protozoal infections. Furthermore, IFNγ is an important activator of macrophages and an inducer of the major histocompatibility complex (MHC) Class II complex molecule expression, whereas IL4 plays a key regulator in humoral and adaptive immunity. Moreover, IL4 induces B-cell class switching to IgE and upregulates MHC Class II production [22,23]. The study results here agree with the results of irradiated viral and bacterial vaccine epitopes reported by Gaidamakova *et al*. [13]. The evaluation of the humoral and cellular immune results demonstrated that the different preparations of irradiated vaccines increased the antibody, IFNγ, and IL4 levels compared with the control and the formalin-killed vaccine. The second dose is an immune booster that gives the vaccine a long-acting immunological efficiency.

The purpose of adding the trehalose during the preparation of the irradiated vaccine was to preserve the bacterial cell protein stability as an important bacterial component responsible for stimulating the immune system to produce a specific immune response. Trehalose has structural and functional roles in protecting bacteria against cold, heat, and oxidative stresses. It is a natural, non-toxic disaccharide (two D-glucose molecules) linked by a glycosidic linkage. It is produced by many invertebrates, a wide variety of plants and algae, bacteria, yeasts, and fungi [24-27]. Trehalose is widely used as a protein stabilizer and cryoprotectant by the food and drug industry [7,27,28]. The result of the antibody level for the trehalose irradiated vaccine was lower than that for the irradiated vaccine without trehalose. The immune efficiency of the trehalose irradiated vaccine results did not agree with results obtained for the bacteria trehalose vaccine [28] and the virus trehalose vaccine [7]. However, Vanaporn and Titball [28] suggested that the role of trehalose protection depends on the type of bacterial cell and high levels of accumulated trehalose delay the bacteria germination of spores. Accordingly, the decrease in the antibody level of G3 (trehalose irradiated vaccine) compared with G2 (irradiated vaccine without trehalose) is likely because of the concentration of trehalose on the metabolic activity of the bacterial cells and thus leads to a decrease in the antibody levels.

To obtain a thermostable vaccine, the immune efficiency of the irradiated vaccine after lyophilization was evaluated. Bacterial cells lyophilization (freeze-drying) is a method established for long-term storage at 30°C to 35°C, and the lyophilized bacterial cells can retain their viability for 10-20 years [29]. Lyophilization of bacterial cells can protect the protein membrane of bacterial cells from destruction during exposure to X-rays and preserve their viability for a long time [22-32]. The results of humoral and cellular immunity produced by trehalose lyophilized irradiated bacterial cell vaccination were at high levels after the second inoculation dose compared with the formalin-killed vaccine and control groups. The results support using the trehalose lyophilized irradiated bacterial cells vaccine, which adds a further advantage to the irradiated vaccine. They are thermostable at 30°C to 35°C and easy to store with the recommendation of decreasing the added percentage of trehalose to 5%.

Overall, the study results agree with the research studies of vaccines developed for microorganisms based on gamma radiation technology. IPVs have broad immune efficiency, and several advantages were reported with increases in the antibody levels on vaccination and after a challenge by microorganisms (*Listeria*, *Foot*-and-*Mouth* Virus, *avian influenza* virus, *influenza* virus, *Salmonella gallinarum*, and *M. haemolytica*) [2-9] and parasites (*human schistosomiasis* and *murine trichinellosis*) [10-12].

## Conclusion

A radiation vaccine is a promising technology that can be used to develop an IPV that provides a broad spectrum of humoral and cellular immunity. This study showed immunological efficiency in rabbits vaccinated with an irradiated *M. haemolytica* vaccine that had higher levels of antibodies, IFNγ and IL4 compared with the group vaccinated with the formalin-killed vaccine. The second dose is an immune booster that provides a long-acting immune efficiency to the vaccine. The lyophilized irradiated bacterial cell vaccine showed promising results that add more advantages to the irradiated vaccine. It is thermostable at 30°C to 35°C and easy to store. Further study is required to optimize the immune efficiency of the irradiated *M. haemolytica* vaccine in sheep as a target animal for *Mannheimia haemolytica*.

## Authors’ Contributions

SA: Carried out the laboratory work and wrote the manuscript. WAN: Carried out the laboratory work related to vaccine preparation. AMM: Carried out the laboratory work related to bacterial isolation and identification. MAEM and WAAM: Carried out the laboratory work on metabolic activity, ELISA, IFNγ, and IL4. All authors have read and approved the final manuscript.
